# Enhancing Machine-Learning Prediction of Enzyme Catalytic Temperature Optima through Amino Acid Conservation Analysis

**DOI:** 10.3390/ijms25116252

**Published:** 2024-06-06

**Authors:** Yinyin Cao, Boyu Qiu, Xiao Ning, Lin Fan, Yanmei Qin, Dong Yu, Chunhe Yang, Hongwu Ma, Xiaoping Liao, Chun You

**Affiliations:** 1 College of Biotechnology, Tianjin University of Science and Technology, Tianjin 300457, China; caoyy@tib.cas.cn (Y.C.);; 2 Tianjin Institute of Industrial Biotechnology, Chinese Academy of Sciences, Tianjin 300308, China; qiuby@tib.cas.cn (B.Q.); ma_hw@tib.cas.cn (H.M.); 3 Department of Life Sciences and Medicine, University of Science and Technology of China, Hefei 230022, China; 4 University of Chinese Academy of Sciences, Beijing 100049, China; 5 National Center of Technology Innovation for Synthetic Biology, Tianjin 300308, China

**Keywords:** optimal catalytic temperature, phosphatase, multiple sequence alignment, conserved amino acids, machine learning

## Abstract

Enzymes play a crucial role in various industrial production and pharmaceutical developments, serving as catalysts for numerous biochemical reactions. Determining the optimal catalytic temperature (*T*_opt_) of enzymes is crucial for optimizing reaction conditions, enhancing catalytic efficiency, and accelerating the industrial processes. However, due to the limited availability of experimentally determined *T*_opt_ data and the insufficient accuracy of existing computational methods in predicting *T*_opt_, there is an urgent need for a computational approach to predict the *T*_opt_ values of enzymes accurately. In this study, using phosphatase (EC 3.1.3.X) as an example, we constructed a machine learning model utilizing amino acid frequency and protein molecular weight information as features and employing the K-nearest neighbors regression algorithm to predict the *T*_opt_ of enzymes. Usually, when conducting engineering for enzyme thermostability, researchers tend not to modify conserved amino acids. Therefore, we utilized this machine learning model to predict the *T*_opt_ of phosphatase sequences after removing conserved amino acids. We found that the predictive model’s mean coefficient of determination (R^2^) value increased from 0.599 to 0.755 compared to the model based on the complete sequences. Subsequently, experimental validation on 10 phosphatase enzymes with undetermined optimal catalytic temperatures shows that the predicted values of most phosphatase enzymes based on the sequence without conservative amino acids are closer to the experimental optimal catalytic temperature values. This study lays the foundation for the rapid selection of enzymes suitable for industrial conditions.

## 1. Introduction

Enzymes, as catalysts for biochemical reactions, play an indispensable role in various biotechnologies, industrial production, and drug development. Accurately predicting the optimal reaction temperature of enzymes helps improve system stability and reaction rates, reduce production costs, and provide critical support for industrial manufacturing. Furthermore, accurately predicting the optimal reaction temperature of enzymes can enable researchers to rapidly screen enzymes suitable for specific processes or experimental conditions, providing robust support for accelerated product development [1].

In the laboratory, accurately determining proteins’ optimal catalytic temperature (*T*_opt_) requires a series of complex procedures. First, substrates related to the specific catalytic activity of the protein must be selected. Then, protein expression and purification are conducted, followed by reactions at different temperatures. The protein’s optimal catalytic temperature is determined by comparing the changes in reaction rates. Due to high costs and long experimental durations, determining the *T*_opt_ of a vast amount of enzymes due to significant advancements in sequencing technology through experimental methods is impractical. Directly predicting the *T*_opt_ of enzymes from sequences would undoubtedly accelerate enzyme screening. With the development of bioinformatics and artificial intelligence [2], researchers have designed various machine-learning models to predict the optimal catalytic temperature of proteins [3]. Among them, the TOME machine learning model developed by Li et al. [4] combines the optimal growth temperature (OGT) of organisms with the sequence information of individual enzymes to predict the enzyme *T*_opt_. This model employs amino acid frequency and OGT data as features and the random forest regression as the model algorithm, achieving a maximum R^2^ of 0.51. However, this study only provides the codes for predicting OGT on the GitHub code repository and does not offer a specific approach for predicting the optimal enzyme catalytic temperature. Subsequently, Gao et al. proposed TOMER [5], which uses the training set of TOME followed by a normal distribution and employs ensemble learning and resampling strategies to increase the R^2^ value to 0.632. Although the model has made progress in predicting the thermal stability of enzymes, it still relies on OGT as supplementary information and cannot directly predict the optimal catalytic temperature of enzymes based solely on sequences. More recently, Engqvist et al. developed a deep-learning-based model, DeepET (https://github.com/EngqvistLab/DeepET_reps) (accessed on 28 March 2024) [6], using optimal growth temperatures (OGT) from 21,498 microorganisms through transfer learning with an R^2^ of 0.57 on the test dataset. Due to the model’s utilization of bacterial growth temperature as the dataset, the predictive performance may not be ideal for certain specific families. However, it is a feasible and reasonable strategy to determine the optimal catalytic temperature of enzymes based on the known *T*_opt_ values of other members within the same enzyme family and then extrapolate to predict the optimal catalytic temperature of enzymes from different families [7]. In this study, we selected phosphatases as our research subject. Phosphatase (EC 3.1.3.X), which catalyzes irreversible dephosphorylation reaction, is often designed as the last step of many in vitro synthetic enzymatic biosystems (ivSEBs) for the production of various chemicals, such as inositol [8,9], glucosamine [10], and allulose, with high product yields [11]. Furthermore, ivSEB often requires thermally stable enzymes because of system stability and easy enzyme purification by heat treatment [9]. Therefore, quickly identifying the *T*_opt_ of phosphatases that meet production conditions becomes crucial in accelerating the industrialization of ivSEB. Therefore, in this study, this enzyme family was used as an example to develop a strategy for the quick prediction of enzyme *T*_opt_. Furthermore, the phosphatase family (EC 3.1.3.X) is a family recorded in the BRENDA database with relatively large records of *T*_opt_ values (Supplementary Data, Table S1).

Conserved amino acids remain unchanged during the long evolutionary process of protein sequences, playing a crucial role in maintaining the basic structure and catalytic function of enzymes [12]. In previous thermal stability studies, researchers have typically utilized non-conserved amino acids to modify enzymes: for instance, Li et al. significantly improved the thermal stability and catalytic efficiency of β-glucuronidase from *Aspergillus terreus* Li-20 by truncation of non-conservative sequences [13]; Xu et al. improved the thermal stability and activity of the thermophilic *Bacillus subtilis* enzyme by substituting non-conservative residues of thermophilic *B. subtilis* enzyme with non-conservative residues of thermophilic *B. subtilis* enzyme [14]; Guo et al. improved the thermal stability of Bs APA aspartic protease via the targeted mutagenesis of non-conserved amino acids near the autocatalytic site region [15]. Therefore, we speculate that conserved and non-conserved amino acids may have varying degrees of influence on enzyme thermostability.

Here, we develop a new machine learning model for predicting the *T*_opt_ of phosphatases (EC 3.1.3.X) based only on the amino acid sequence, with the potential to be generalized to predict the *T*_opt_ of other enzymes from different families. We obtained phosphatase information from the BRENDA and UniProt databases and employed multiple sequence alignments to remove the conserved amino acids from the sequences. Subsequently, we computed multiple features for each phosphatase sequence and attempted to establish a machine-learning prediction model for the *T*_opt_ of phosphatase based on both wild-type and non-conserved amino acid sequence information. Compared to the wild-type sequences, the predicted *T*_opt_ values obtained from the sequences without conserved amino acids are generally closer to the experimentally determined values. To further validate the reliability of the model, we collected 10 phosphatases with undetermined optimal catalytic temperatures for experimental validation. The results also indicate that the predicted values of nine phosphatases based on the sequence without conserved amino acids are closer to the experimental values, further confirming the reliability of our model. The strategy developed in this study may provide a generalized protocol for accelerating the screening process of suitable enzymes that will be utilized in harsh environments.

## 2. Results

### 2.1. Phosphatase Data Collection and Processing

**Figure 1:**
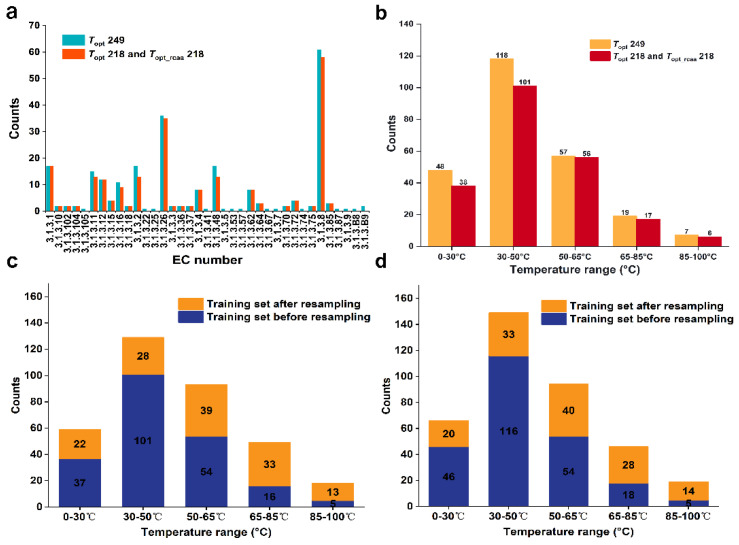
(**a**). Distribution of phosphatase under different EC groups in our defined datasets. (**b**). Distribution of phosphatase datasets across different temperature ranges. (**c**). Distribution of the *T*_opt_249 phosphatase training dataset. (**d**). Distribution of the *T*_opt_218 and *T*_opt_rcaa_218 phosphatase training datasets.

We obtained a dataset containing 249 phosphatases with experimentally validated *T*_opt_ values from the BRENDA and UniProt databases. This dataset is referred to as the *T*_opt_249 dataset (Supplementary Data, Table S2). We grouped these phosphatases based on the fourth digit of their EC numbers, resulting in 37 groups. After removing sequences with lengths less than 50 and groups containing only one enzyme, 234 data points remained (23 groups). Due to the small number of conserved amino acids in the groups with EC numbers 3.1.3.2, 3.1.3.8, 3.1.3.11, 3.1.3.16, 3.1.3.26, and 3.1.3.48, we constructed sequence similarity networks (SSNs) (Supplementary Material, Figure S1) for each group and removed 14 clusters that contained only a single node in the similarity network. The dataset was then reduced to 220 data points. This process allowed us to determine the conserved amino acids for each group (Supplementary Data, Table S3) and remove these conserved amino acids from the original sequences to generate a new set of sequences. Due to the sequence length being less than 50 after removing conserved amino acids for the group with the EC number 3.1.3.B9, we excluded two data points from this group. In the end, we obtained 22 groups containing 218 phosphatases without conserved amino acids, which formed the *T*_opt_rcaa_218 dataset (Supplementary Data, Table S4). To compare prediction accuracy, these 218 phosphatases with the original sequence formed another dataset named *T*_opt_218 (Supplementary Data, Table S5). We conducted a statistical analysis of the phosphatase counts under different EC groups in the datasets of *T*_opt_249, *T*_opt_rcaa_218 and *T*_opt_218. Among them, the group with EC number 3.1.3.8 has the highest number of phosphatases, with 61 in the *T*_opt_249 dataset and 58 in the *T*_opt_218 and *T*_opt_rcaa_218 datasets. Secondly, the group with EC number 3.1.3.26 has 36 phosphatases in the *T*_opt_249 dataset and 35 in the *T*_opt_218 and *T*_opt_rcaa_218 datasets (Figure 1a).

We divided the *T*_opt_218 and *T*_opt_rcaa_218 datasets into five temperature ranges based on their measured *T*_opt_ values: [0–30 °C, 30–50 °C, 50–65 °C, 65–85 °C, 85–100 °C] (Figure 1b). We found that about 50% of enzymes were in the 30–50 °C range, while less than 3% were in the 85–100 °C range. To ensure that data for each temperature band is included in both the training and test sets, half of the data in the smallest set (85–100 °C) is used for training. In the end, we selected two data points from each of the five temperature ranges, totaling 10 data points, to form the test set. The remaining data was used for the training set. (See Section 3.3)

In this study, the *T*_opt_ of phosphatases exhibit a normal distribution. This imbalanced distribution may lead to insufficient sampling in temperature regions with fewer data points, thereby affecting prediction outcomes and reducing the overall model performance [16,17,18]. To address the issue of data imbalance, we referenced the study by Gado et al. [5], who resolved the problem of uneven distribution in the training set of the TOME model through resampling strategies, thus improving the predictive accuracy of the TOME model. Therefore, before fitting regression variables each time, we applied a regression resampling strategy to the training dataset, ensuring that the quantity of data contained in the rare domain is relatively balanced with the normal domain. By applying the resampling strategy to the training set of *T*_opt_249 (Supplementary Data, Table S6), a significant decrease in the number of temperature ranges with rich data distribution was observed: reduced to one-fourth of the original. The number of temperature ranges with less data distribution significantly increased, approximately tripled compared to the original (Figure 1c). Additionally, applying the resampling strategy to the training set of *T*_opt_218 and *T*_opt_rcaa_218 resulted in similar effects (Figure 1d), thereby achieving a relatively balanced distribution of data across all temperature ranges within the entire training set.

### 2.2. Prediction of Optimal Catalytic Temperature for Phosphatases by Previous Tools

We first used the deep neural network model DeepET developed by Engqvist et al. [6] to predict the above *T*_opt_249 and *T*_opt_218 datasets of phosphatase enzymes. On the *T*_opt_249 phosphatase dataset, the model estimated an R^2^ of 0.372 (Supplementary Material, Figure S2a), and on the *T*_opt_218 phosphatase dataset, the model estimated an R^2^ of 0.358 (Supplementary Material, Figure S2b). It is evident that the DeepET model does not achieve satisfactory accuracy in predicting the optimal catalytic temperature for the phosphatase enzyme family.

Later, we employed the TOMER model developed by Gado et al. [5] to predict the Topt249 and Topt218 datasets of phosphatase enzymes. As this model requires the growth temperature of bacteria as auxiliary information, we obtained the bacterial growth temperature information from DSMZ (https://webshop.dsmz.de/index.php) (accessed on 28 March 2024). For the *T*_opt_249 dataset, only 116 data points had corresponding bacterial growth temperature information, and we used the TOMER model to predict these 116 data points (*T*_opt_116) (Supplementary Data, Table S7), resulting in an R² value of 0.522 (Supplementary Material, Figure S3a). For the *T*_opt_218 dataset, only 100 data points had corresponding bacterial growth temperature information. Similarly, we predicted these 100 data points (*T*_opt_100) (Supplementary Data, Table S8), resulting in an R² value of 0.47 (Supplementary Material, Figure S3b).

Overall, DeepET’s performance in predicting phosphatases is not ideal. Although TOMER shows some improvement in accuracy compared to DeepET, it still cannot predict enzymes without bacterial growth temperature information. This result indicates that it is necessary to develop an alternative model with better accuracy in predicting the *T*_opt_ of enzymes.

### 2.3. Feature Extraction and Model Evaluation Based on T_opt_249

**Figure 2:**
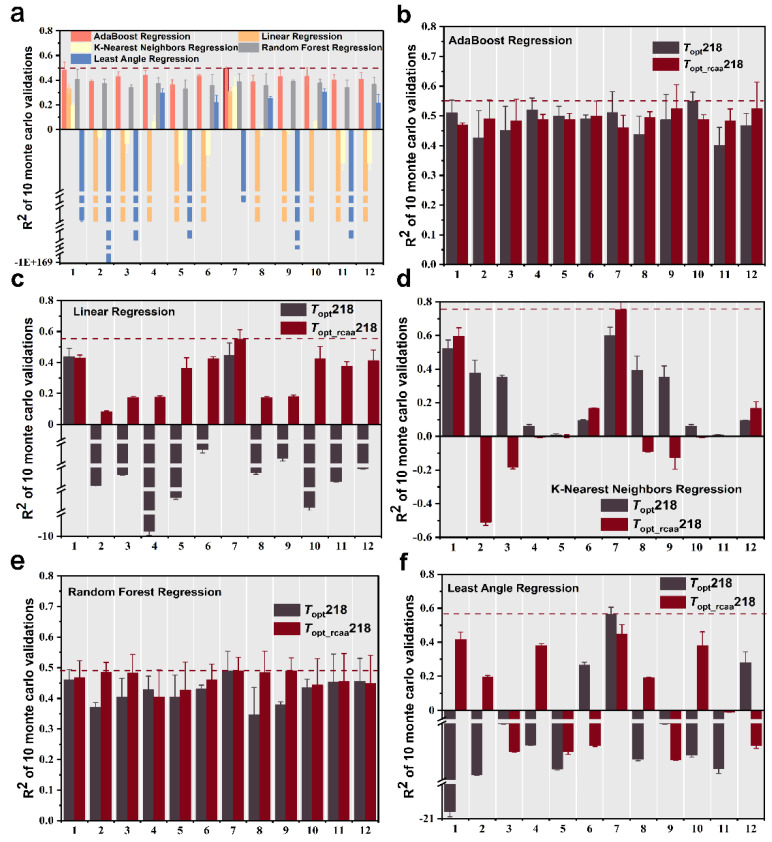
Ten Monte Carlo cross-validation coefficient of determination (R^2^) results for five regression models fitted with different combinations of features in different datasets. The error bars represent the standard deviation of the R^2^ scores. R^2^ values for five regression models fitted with different combinations of features in *T*_opt_249 dataset (**a**), R^2^ values for AdaBoost regression machine learning algorithm (**b**), linear regression machine learning algorithm (**c**), K-nearest neighbors regression machine learning algorithm (**d**), random forest regression machine learning algorithm (**e**), and least angle regression machine learning algorithm (**f**) fitted with different combinations of features in *T*_opt_218 and *T*_opt_rcaa_218 datasets. Features 1–12 are listed in Table 1 for details.

We extracted four sets of features from the phosphatase sequences, including amino acid frequency, dipeptide frequency, protein molecular weight [19], and protein descriptors (conjoint triad features [20] and distribution descriptors [21]). We trained and tested every feature as well as combinations of these features (total 12 sets of feature and feature combinations, Table 1) using five different machine learning regression models to predict the *T*_opt_ of phosphatase. First, we computed the multiple sets of features for the phosphatase data in the *T*_opt_249 dataset and evaluated the predictive performance based on the mean coefficient of determination (R^2^) from ten-fold Monte Carlo cross-validation After comparing all combinations of features and algorithms, we found that using the combination of amino acid frequency and protein molecular weight as features, along with the AdaBoost Regression machine learning algorithm for prediction, resulted in the highest prediction accuracy, albeit only 0.495 (Figure 2a).

### 2.4. Removing the Conserved Amino Acid in the Protein Sequence Facilitates the Prediction of Phosphatase Catalytic Temperature Optima by Machine-Learning

To validate our hypothesis regarding the potentially different impacts of conservative and non-conservative amino acids on enzyme thermal stability, subsequently, we also computed the 12 sets of features for the *T*_opt_218 and *T*_opt_rcaa_218 phosphatase datasets. We then fitted the same five machine learning regression models mentioned above, adjusting parameters to optimize the models. The performance of the different regression algorithms was evaluated using the same evaluation methodology described above, and in the case of negative values when fitting the algorithms for some features, we also set breakpoints to better represent all the R^2^ values.

We initially employed the AdaBoost regression machine learning algorithm, combined with the 12 different feature sets from Table 1, to predict the optimal catalytic temperature of phosphatases. We observed that when considering the five feature sets (1, 4, 5, 7, 10) listed in Table 1, the R^2^ values based on complete sequence were higher than those based on the sequence without conserved amino acids, while R^2^ values were higher based on the sequence without conserved amino acids for the other seven feature sets. However, the highest predictive accuracy (R^2^ = 0.55) was achieved based on the complete sequence and employed the feature combination from the 10th group in Table 1 (Figure 2b).

Using the linear regression machine learning algorithm for prediction, it was found, only using the 1st group in Table 1 as a feature, that the R^2^ value based on the complete sequence was higher than that based on the sequence without conserved amino acids, while R^2^ values based on the sequence without conserved amino acids were all higher than those based on the complete sequence. In addition, most of the R^2^ values based on the complete sequence were negative. When employing the feature of the 7th group in Table 1 to predict the *T*_opt_ based on sequence without conserved amino acids, it achieved the highest predictive accuracy with an R^2^ value of 0.55 on the test set (Figure 2c).

Using the K-nearest neighbors regression machine learning algorithm, we observed that only when considering the eight feature sets listed in Table 1 (2, 3,4, 5, 8, 9, 10, 11), the prediction based on the complete sequence showed higher accuracy compared to the scenario where conserved amino acids were excluded. However, the best-performing prediction occurred based on the sequence without conserved amino acids, with the 7th group of features from Table 1 achieving the highest R^2^ prediction accuracy of 0.755 on the test set (Figure 2d).

When using the random forest regression machine learning algorithm, we found that most of the predictions based on sequences without conserved amino acids are more accurate than those based on the complete sequence. The best-performing model utilized only non-conservative amino acid sequences with the feature combination from the 9th group of Table 1, achieving the highest R^2^ prediction accuracy of 0.49 on the test set (Figure 2e).

Using the least angle regression machine learning algorithm, when considering the 6th, 7th, and 12th feature sets in Table 1, the model performs better based on the complete sequence than the sequence without conserved amino acids. The optimal model utilized the complete sequence and employed the 7th feature set from Table 1. It achieved the highest predictive accuracy with an R^2^ of 0.566 on the test set (Figure 2f).

Throughout the entire training process, we observed that for both the complete sequence and the sequence with conserved amino acids removed, most models demonstrated higher predictive accuracy with the sequence excluding conserved amino acids. The best of these prediction models were all trained by the K-nearest neighbor regression algorithm using a combination of amino acid frequencies and protein molecular weight features. Compared to the best-predicting accuracy based on a complete sequence (R^2^ = 0.599), this optimal model improves the prediction accuracy to 0.755 based on the sequence without conserved amino acids (Wilcoxon test, *p*-value = 0.006), which is significantly higher than the accuracy of the complete sequence. 

Subsequently, we utilized the combination of amino acid frequency and protein molecular weight to fit the K-nearest neighbors’ regression algorithm, predicting the *T*_opt_ of unmeasured phosphatases by removing conserved amino acids.

### 2.5. Prediction of T_opt_ of Unmeasured Phosphatases by Optimized Machine Learning Model and Experimental Validation

**Figure 3:**
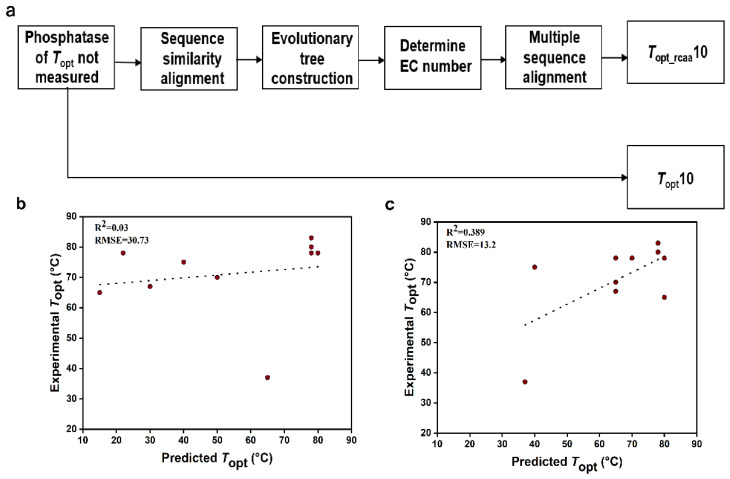
Phosphatase data processing and comparison of predicted and experimental values for *T*_opt_10 and *T*_opt_rcaa_10 Datasets. RMSE: root mean square error. (**a**). Processing workflow for predicting the *T*_opt_ of unknown phosphatase. (**b**). Comparison between predicted *T*_opt_ values and experimental *T*_opt_ values for the *T*_opt_10 phosphatase dataset. (**c**). Comparison between predicted *T*_opt_ values and experimental *T*_opt_ values for the *T*_opt_rcaa_10 phosphatase dataset.

We randomly selected 10 phosphatases with UniProt IDs of O58216, Q5SLK1, E8UU74, Q97C22, Q72I84, Q5SKD4, A0LR15, Q8Z989, O59374, and Q5SI65 (Table 2). The dataset comprising the complete sequences of these 10 phosphatases was named *T*_opt_10 (Supplementary Data, Table S9). These phosphatases have not been experimentally determined for *T*_opt_. The specific process for handling the above phosphatase data is illustrated in Figure 3a. Firstly, we need to determine the EC numbers of these phosphatases. We constructed 10 phylogenetic trees by aligning the sequences of these phosphatases with the sequences in the model training set (Supplementary Material, Figure S4). Based on the EC numbers of phosphatase sequences with relatively short evolutionary distances to the target phosphatase, we determined the EC number of the target phosphatase (Table 2). Subsequently, the target sequence was aligned with sequences from the same EC number, and conservative amino acids were removed from the target sequence (Supplementary Data, Table S10). Thus, the other dataset (*T*_opt_rcaa_10) was obtained, consisting of the 10 sequences with conservative amino acids removed (Supplementary Data, Table S11). Next, we extracted the amino acid frequency and protein molecular weight information from the phosphatase sequences and then utilized the K-nearest neighbors regression algorithm to predict the *T*_opt_ (Table 2) of phosphatase sequences in the two datasets (*T*_opt_10 and *T*_opt_rcaa_10). Meanwhile, through experiments, the experimental *T*_opt_ values for the 10 phosphatases were determined to be 78 °C, 80 °C, 75 °C, 65 °C, 83 °C, 78 °C, 67 °C, 37 °C, 78 °C, and 70 °C (Supplementary Material, Figure S5), respectively. By comparing the predicted values with the experimental values, we calculated the root mean square error (RMSE) and R-square (R^2^). We observed that the prediction results based on the sequence without conserved amino acids (R^2^ = 0.389, RMSE = 13.2) were significantly better than those based on the complete sequence of phosphatases (R^2^ = 0.03, RMSE = 30.7) (Figure 3). 

Interestingly, we observed that the 10 randomly selected test samples predominantly originated from EC numbers with fewer training samples (e.g., EC 3.3.3.102, 3.3.3.104, 3.3.3.70, 3.3.3.3, 3.3.3.18), posing a challenge to our model. This task was more difficult compared to selecting samples from EC numbers with a larger sample size. The test results of the final 10 samples indicated that our approach still maintained a reasonable level of prediction accuracy, demonstrating the model’s good generalization capability.

## 3. Materials and Methods

### 3.1. Prediction Model for Optimal Catalytic Temperature of Phosphatases

**Figure 4:**
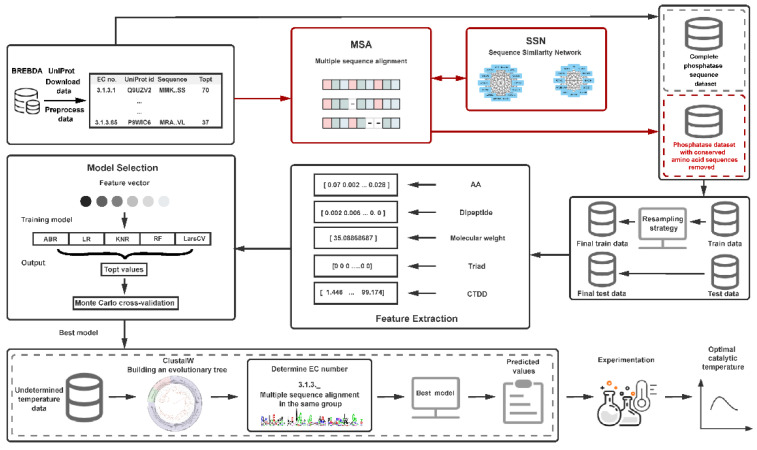
Schematic overview of the machine learning model for predicting the optimal catalytic temperature of phosphatases. AA: amino acid frequencies. Dipeptide: dimer frequencies. Molecular Weight: protein molecular weight. Triad: conjoint triad features. CTDD: distribution descriptor.

The basic process of this research work includes the following six steps. (1) Data collection and processing: Download phosphatase data from the BRENDA and UniProt databases, group by the fourth digit of the EC number for multiple sequence alignment, and remove conserved amino acids. The issue of too few conserved amino acids in the group was addressed by constructing a sequence similarity network (SSN). Finally, datasets containing sequences with conserved amino acids were removed, and complete sequences were obtained. (2) Training set and test set division: The dataset was divided into five temperature ranges. From the temperature range containing the least data, 1/4 of the data was taken, and an equal amount was taken from each of the other temperature ranges to form the test set. The remaining data was used as the training set. Before each feature extraction, the training set was resampled. (3) Feature extraction and selection: Multiple features including amino acid frequency, dipeptide frequency, protein molecular weight, and protein descriptors (conjoint triad features and distribution descriptors) were computed for each phosphatase sequence. (4) Model selection and training: Various machine learning algorithms were fitted with different feature combinations, and parameter tuning was conducted. (5) Model evaluation: The performance of the prediction models was assessed using ten-fold Monte Carlo cross-validation. (6) Experimental validation: Predictions were made for the unmeasured experimental values of phosphatase sequences using the optimal model, followed by experimental validation. The framework flowchart is illustrated in Figure 4.

### 3.2. Dataset Construction

#### 3.2.1. Construction of the Dataset for Wild-type Sequences of Phosphatases

In this study, we obtained phosphatase data from the BRENDA (https://www.brenda-enzymes.org) (accessed on 15 March 2022) [22] and UniProt (https://www.UniProt.org/) (accessed on 15 March 2022) [23] databases. Firstly, we developed a custom Python script [24] to extract the EC numbers (EC 3.1.3.X) of 113 phosphatases from the webpage of the BRENDA database (https://www.brenda-enzymes.org/all_enzymes.php) (accessed on 15 March 2022). Subsequently, we constructed a URL (https://www.brenda-enzymes.org/enzyme.php?ecno=) (accessed on 17 March 2022) based on these EC numbers and retrieved and saved the information web pages of the phosphatases using custom scripts for the application programming interface. We parsed the web pages of the phosphatases using custom scripts, analyzed the page content, and extracted the UniProt ID and temperature information of the phosphatases from the ‘TEMPERATURE OPTIMUM’ table. Phosphatases without temperature records were directly removed. Finally, we constructed a URL (https://www.uniprot.org/uniprotkb) (accessed on 20 March 2022) fiusing the UniProt ID of the phosphatases to retrieve the corresponding amino acid sequences in FASTA format from the UniProt database. After removing duplicate phosphatases, we obtained a total of 249 phosphatases from Brenda and UniProt.

#### 3.2.2. Construction of Phosphatase Dataset with Conserved Amino Acids Removed

Divide the obtained phosphatase data into groups based on the fourth digit of the EC number. By calling the ClustalOmegaCommandlinemethod from the Bio.Align.Applications.ClustalOmegaCommandline module and setting the similarity threshold to threshold = 1, we conducted a multiple sequence alignment of the phosphatases within the same group [25,26]. This method inserts gaps in shorter sequences during the alignment process to ensure that the number of amino acids in the protein sequences are equal, generating intermediate FASTA files for sequence alignment. Subsequently, we used the AlignIO.readmethod from Biopython to read this FASTA file, thereby obtaining the UniProt ID corresponding to the aligned sequences, the position indices of conserved amino acids in the original sequence, and the respective information of these conserved amino acids. Finally, we removed the corresponding conserved amino acids based on the UniProt ID of the phosphatase and the position indices of conserved amino acids in the original sequence.

Based on the grouping, we found that some groups contained only one phosphatase. Since multiple sequence alignment could not be performed in such cases, we removed these entries from the dataset. At the same time, some groups contained two phosphatase data, where the number of remaining amino acids after removing the conserved amino acids from the original sequence was less than 50. We removed these sequences from the dataset to ensure the training set’s quality and accuracy. In the end, a total of 17 data points were removed.

On the other hand, within the groups with a larger number of enzymes, as the number of aligned sequences increases, the number of conserved amino acids also decreases significantly. In these groups with a large number of enzymes, we utilized the ENZYME FUNCTION INITIATIVE TOOLS (EFI) online network tool (http://efi.igb.illinois.edu/efi-est/) (accessed on 7 October 2022) [27] to construct a sequence similarity network (SSN) [28]. We further grouped and aligned the sequences based on gene clusters. First, we compiled the phosphatase enzyme sequences from the same group into a FASTA file and uploaded it to EFI. We then conducted a protein sequence similarity analysis using the default parameters. Based on the results returned by the website, we adjusted the similarity threshold (Supplementary Material, Table S1) to obtain a sequence similarity network with the fewest isolated nodes and generated the corresponding XGMML file.

Subsequently, we visualized the generated sequence similarity network XGMML file using Cytoscape (V 3.10.0). In the network, each node represents a protein, and the corresponding UniProt ID is displayed on the node. We then used each cluster of enzymes within the network as a new sub-group, followed by sequence alignment and removal of conserved amino acids. Clusters with only one node in the sequence similarity network were removed, resulting in a total of 14 data points being eliminated.

Due to the removal of some phosphatase enzymes during the sequence alignment process, to ensure that the training and test sets are the same before and after removing conserved amino acids, we constructed a dataset containing the complete sequences identical to the dataset without conserved amino acids. Each dataset contains 218 phosphatase enzyme entries.

### 3.3. Training and Testing Set Splitting

The data of optimal catalytic temperatures for phosphatases follow a normal distribution, albeit with an imbalance, and the majority of phosphatases exhibit optimal catalytic temperatures around 37 °C. We analyzed the temperature information in the dataset and divided the entire dataset into five sets based on the target value (*T*_opt_): [0–30 °C, 30–50 °C, 50–65 °C, 65–85 °C, 85–100 °C] [5]. Then, we counted the number of data contained in these five sets. From the set with the fewest data points (85–100 °C), we selected 1/4 of the samples. The same number of samples was selected from each of the other sets to form the test set, and the remaining data was used for the training set. 

To mitigate the issue of phosphatase data imbalance, we applied the regression resampling strategy proposed by Gado et al. [5] to the training set. By utilizing the random_oversamplemethod from the resreg package, with parameters cl = 21 and ch = 64, we divided the data with temperatures less than 21 °C and greater than 64 °C into the rare domain, while the remaining data constituted the normal domain. Then, by setting the ‘relevance_threshold’ parameter to its default value of 0.5, we randomly oversampled half of the samples in the rare domain, ensuring that after sampling, the rare domain contained an equal number of phosphatases as the normal domain.

### 3.4. Feature Extraction and Selection

We computed multiple sets of features for each protein. Firstly, we computed a set of 1 × 20-dimensional amino acid frequencies (AA) and a set of 20 × 20-dimensional dipeptide frequencies (Dipeptide).

The formula for calculating amino acid frequency is as follows:

(1) $$f(t)=\frac{N(t)}{N},t\in \left\{A,C,D,\ldots ,Y\right\}$$

*N* is the number of amino acids in the protein sequence, and *t* is some natural amino acid in the sequence. 

The formula for calculating dipeptide frequency is as follows:

(2) $$F_{rs}=\frac{N_{rs}}{N-1},r,s\in \left\{A,C,D,\ldots ,Y\right\}$$

where *N*_rs_ represents the number of occurrences of dipeptide *rs* in the protein sequence, and *N* represents the total length of the protein sequence.

In addition, we analyzed the phosphatase sequences using the ProteinAnalysis method from the Bio.SeqUtils.ProtParam module in the Biopython library [29] and obtained a set of 1 × 1-dimensional protein molecular weights by invoking the molecular_weightmethod. We then computed two sets of protein descriptors using the PyDPI (V1.0) Python toolkit [30,31]. One set is a 1 × 343-dimensional conjoint triad feature (CTF) obtained by analyzing the phosphatase enzyme sequence using the GetProDesmethod and then invoking the GetTriadmethod. The other set is a 1 × 105-dimensional distribution descriptor (CTDD) obtained by invoking the CalculateD method. We combined single and multiple feature sets and utilized the StandardScalerclass from the scikit-learn (V 0.24.1) package to generate a StandardScalerobject. We then standardized the feature combinations by calling the transformmethod. Then, we fitted five different machine learning algorithms for training and testing [32].

### 3.5. Construction and Training of Machine Learning Models

In this study, we utilized various regression algorithms available in the Python machine learning package Scikit-learn (V 0.24.1), including linear regression, random forest regression, K-nearest neighbors regression, AdaBoost regression, and least angle regression (Lars CV), to construct suitable regression models for predicting the optimal catalytic temperature of phosphatases.

We employed the ordinary least squares linear regression method in multiple linear regression. For the random forest regression algorithm, we adjusted the number of trees grown and tested different values of n_estimators (ranging from 10 to 100), while keeping other parameters at their default values. In the K-nearest neighbors regression, we considered both uniform weighting and Euclidean distance and tested different values of K (ranging from 1 to 15). We ensemble 50 regression trees as base regression models for the AdaBoost regression algorithm, with each tree considering at least two instances per leaf. As for the Lars CV regression algorithm, we set it up using default parameters.

### 3.6. Evaluation of Machine Learning Models

Because the data follows a normal distribution, random splitting of the data would still result in an imbalanced distribution between the training and testing sets, leading to performance metrics (R^2^) being overly weighted towards frequently occurring data and failing to adequately capture performance in the tails of the distribution. Therefore, when evaluating the performance of the regressors, we did not employ traditional k-fold cross-validation techniques. Instead, we utilized 10 iterations of Monte Carlo cross-validation (MCCV) [33] on a nearly uniformly distributed test set. The dataset was divided into training and testing sets ten times. Then, by computing the coefficient of determination (R^2^) between the predicted and actual values of the test set over ten iterations, the average R^2^ was calculated to evaluate the model’s predictive performance.

### 3.7. Enzyme Expression and Purification

To further validate the reliability of our model, based on the growth temperatures of the bacteria, we randomly selected nine phosphatases from bacteria with relatively high growth temperatures and one phosphatase from bacteria with a lower growth temperature (The UniProt IDs are O58216, Q5SLK1, E8UU74, Q97C22, Q72I84, Q5SKD4, A0LR15, Q8Z989, O59374, and Q5SI65) from the study by Huang et al. [34]. These enzymes all lack experimental measurements for *T*_opt_. The genes of the aforementioned 10 phosphatases were synthesized and subcloned into the pET28a vector by Suzhou Geneweave Biotech Co., Ltd. (Suzhou, China). Subsequently, the pET28a plasmids containing the target genes were transformed into Escherichia coli TOP10 for plasmid amplification. Subsequently, the plasmids were extracted and transformed into *E. coli* BL21(DE3) for the expression of phosphatases. The recombinant strains containing the plasmids were cultured in a Luria-Bertani medium at 37 °C. When the OD600 of the culture reached 0.8–1.2, gene expression was induced by adding isopropyl β-D-1-thiogalactopyranoside (IPTG) to a final concentration of 100 μM, followed by further incubation at 16 °C for 18 h. The culture was then centrifuged at 4000× *g* for 30 min to collect the cells. The enzymes were purified using the Ni-NTA method, and the purity of the proteins was assessed via sodium dodecyl sulfate-polyacrylamide gel electrophoresis (SDS-PAGE). 

### 3.8. Determination of Enzyme Activity and Topt of Phosphatases

The enzyme at a certain concentration was incubated with 1 mM para-nitrophenyl phosphate in a 100 mM Tris-HCl buffer (pH 7.4) containing 5 mM MgCl_2_ at different temperatures (37–80 °C) for 10 min. The activity of the phosphatase was determined by measuring the amount of para-nitrophenol produced. One unit of phosphatase activity is defined as the amount of enzyme required to release 1 μmol of para-nitrophenol per minute [35]. The absorbance of the product para-nitrophenol at 405 nm was monitored in real-time using a UV-visible spectrophotometer (model: Evolution one). We used bovine serum albumin as the standard and determined the protein concentration using the Bradford method [36]. The temperature at which the enzyme activity was 100% is defined as *T*_opt_, with the maximum activity measured for a single enzyme considered as 100%. The enzyme activity at other temperatures can then be expressed as relative enzyme activity relative to the maximum activity.

### 3.9. Determining the EC Number of Phosphatase Enzymes

We utilized the ClustalW online tool (https://www.genome.jp/tools-bin/clustalw) (accessed on 5 July 2022) [37,38,39] to perform sequence similarity alignment between the sequences of the 10 phosphatase enzymes without annotated optimal catalytic temperatures and the sequences in the model training dataset, obtaining the alignment results in DND format. We employed the MEGA11 software (V 11.0.13) to visualize the DND files containing the alignment results, constructing 10 evolutionary trees. We determined the position of the target phosphatase in the evolutionary tree topology using its UniProt ID and selected phosphatases with relatively shorter evolutionary distances from the target phosphatase. Based on the EC numbers of the related phosphatases, we determined the EC number of the target phosphatase.

## 4. Discussion

In this study, we proposed a tool that solely relies on protein sequences, without additional experimental conditions, to directly predict the *T*_opt_ of phosphatases. This tool can assist researchers in rapidly selecting phosphatases suitable for specific processes or experimental conditions. 

In model development, we discovered that a basic feature set with the least number of features combined with the KNN algorithm provided superior prediction outcomes. This success is partly due to the significance of amino acid frequency, supported by Li et al.s’ [4] research, which identified amino acid frequency and bacterial growth temperature as key features with the former accounting for half of the model’s performance. In this study, we found that, in addition to amino acid ratio, molecular weight is a potent predictor for KNN, substantially enhancing accuracy (R² rose from 0.46 to 0.755). This may be because KNN excels in identifying similar neighborhoods in low-dimensional spaces with simple features, leading to more precise predictions.

Additionally, it is interesting to note that by removing conserved amino acids from the protein sequences, we achieved better accuracy in predicting the optimal catalytic temperature of phosphatases. Removing conserved amino acids improves the model’s predictive performance (R^2^) by approximately 15%. Finally, we used the optimal model to predict the *T*_opt_ of some unknown phosphatases. The results show that removing conserved amino acids reduced RMSE by approximately 57.04% from 30.7 to 13.2. Therefore, we speculate that conserved and non-conserved amino acids may have varying degrees of influence on enzyme thermostability. This study also lays the groundwork for utilizing machine learning to predict the optimal catalytic temperature of other enzyme families by removing conserved amino acids from their sequences. 

Due to the limited number of reported optimal catalytic temperatures for phosphatases in the literature, this study faced the challenge of having a relatively small dataset for training the model. Furthermore, there is currently a lack of research findings regarding highly correlated features with the optimal catalytic temperature of enzymes. If more experimental data and richer feature information were available, such as from aspects like secondary structure content [40], three-dimensional structures [41], and the physicochemical properties of amino acids [42], we hypothesize that the predictive accuracy of *T*_opt_ could be further improved. In this study, we aimed to predict the optimal catalytic temperature solely based on sequence information as it could help us screen the desired enzyme quickly. In future work, we will consider predicting *T*_opt_ based on the 3-D structure of enzymes using AI tools, such as AlphaFold, as well as other features related to the optimal catalytic temperature of the enzyme. On the other hand, we will actively gather more optimal catalytic temperature data from other enzyme families. Applying our validated approach of removing conserved amino acids from protein sequences significantly improves the accuracy of machine-learning predictions for the optimal catalytic temperature of enzymes. This conclusion guides us in developing prediction tools for the *T*_opt_ of other enzyme families.

## 5. Availability of Final Model, Data, and Code

All phosphatase sequence files and related datasets can be found in the GitHub repository at https://github.com/cyinyin/Tpho/tree/main/data (accessed on 28 March 2024). This includes the *T*_opt_249, *T*_opt_218, and *T*_opt_rcaa_218 datasets. Additional *T*_opt_10 and *T*_opt_rcaa_10 datasets for evaluating model performance can be found in the GitHub repository at https://github.com/cyinyin/Tpho/tree/main/data/Experimentalvalidationdata (accessed on 28 March 2024).

## Figures and Tables

**Table 1:** A summary of different feature combinations and dimensions generated from enzyme sequences used in this study.

No.	Feature Group	Number of Descriptors
1	amino acid frequencies	20
2	dipeptide frequencies	400
3	amino acid frequencies and dipeptide frequencies	420
4	amino acid frequencies, protein descriptors (conjoint triad features and distribution)	468
5	dipeptide frequencies, protein descriptors (conjoint triad features and distribution)	848
6	amino acid frequencies, dipeptide frequencies, protein descriptors (conjoint triad features and distribution)	868
7	amino acid frequencies, protein molecular weights	21
8	dipeptide frequencies, protein molecular weights	401
9	amino acid frequencies, dipeptide frequencies, protein molecular weights	421
10	amino acid frequencies, protein molecular weights, protein descriptors (conjoint triad features and distribution)	469
11	dipeptide frequencies, protein molecular weights, protein descriptors (conjoint triad features and distribution)	849
12	amino acid frequencies, dipeptide frequencies, protein molecular weights, protein descriptors (conjoint triad features and distribution)	869

**Table 2:** Predicted and experimental *T*_opt_ of undetermined phosphatases.

No.	UniProt ID	EC Number	*T*_opt_10 Predicted *T*_opt_ (°C)	*T*_opt_rcaa_10 Predicted *T*_opt_ (°C)	Experimental *T*_opt_ (°C)
1	O58216	3.1.3.102	80	80	78
2	Q5SLK1	3.1.3.104	78	78	80
3	E8UU74	3.1.3.3	40	40	75
4	Q97C22	3.1.3.70	15	80	65
5	Q72I84	3.1.3.102	78	78	83
6	Q5SKD4	3.1.3.70	78	65	78
7	A0LR15	3.1.3.12	30	65	67
8	Q8Z989	3.1.3.18	65	37	37
9	O59374	3.1.3.104	22	70	78
10	Q5SI65	3.1.3.102	50	65	70

## Data Availability

The standalone version and related data are provided at https://github.com/cyinyin/Tpho (accessed on 28 March 2024).

## Supplementary Materials

The following supporting information can be downloaded at: https://www.mdpi.com/article/10.3390/ijms25116252/s1.
