# Supplementary material for: Enhancing Machine-Learning Prediction of Enzyme Catalytic Temperature Optima through Amino Acid Conservation Analysis
Source: Int J Mol Sci. 2024 Jun 6;25(11):6252. doi: 10.3390/ijms25116252 (PMC11173260; doi:10.3390/ijms25116252)
Supplement: Supplementary file 1 [file ijms-25-06252-s001.zip › Supplementary Material.pdf]

Supplementary Material

Enhancing Machine-Learning Prediction of Enzyme Catalytic Temperature Optima through Amino Acid Conservation Analysis

Yinyin Cao[1,2], Boyu Qiu[2,3], Xiao Ning[2,4], Lin Fan[2,4], Yanmei Qin[2,4], Dong Yu[1,2], Chunhe Yang[1,2], Hongwu Ma[2,5], Xiaoping Liao[2,5*], and Chun You[2,4,5*]

[1] College of Bioengineering, Tianjin University of Science and Technology, Tianjin, 300457, China

[2] Tianjin Institute of Industrial Biotechnology, Chinese Academy of Sciences, Tianjin, 300308, China

[3] Department of Life Sciences and Medicine, University of Science and Technology of China, Hefei 230022, People's Republic of China

[4]University of Chinese Academy of Sciences, Beijing, 100049, China

[5] National Center of Technology Innovation for Synthetic Biology, Tianjin, 300308, China

*Corresponding author: Xiaoping Liao, Phone: (+86)-022-24828758, Email: liao_xp@tib.cas.cn

Chun You, Phone: (+86)-022-24828795, Email: you_c@tib.cas.cn

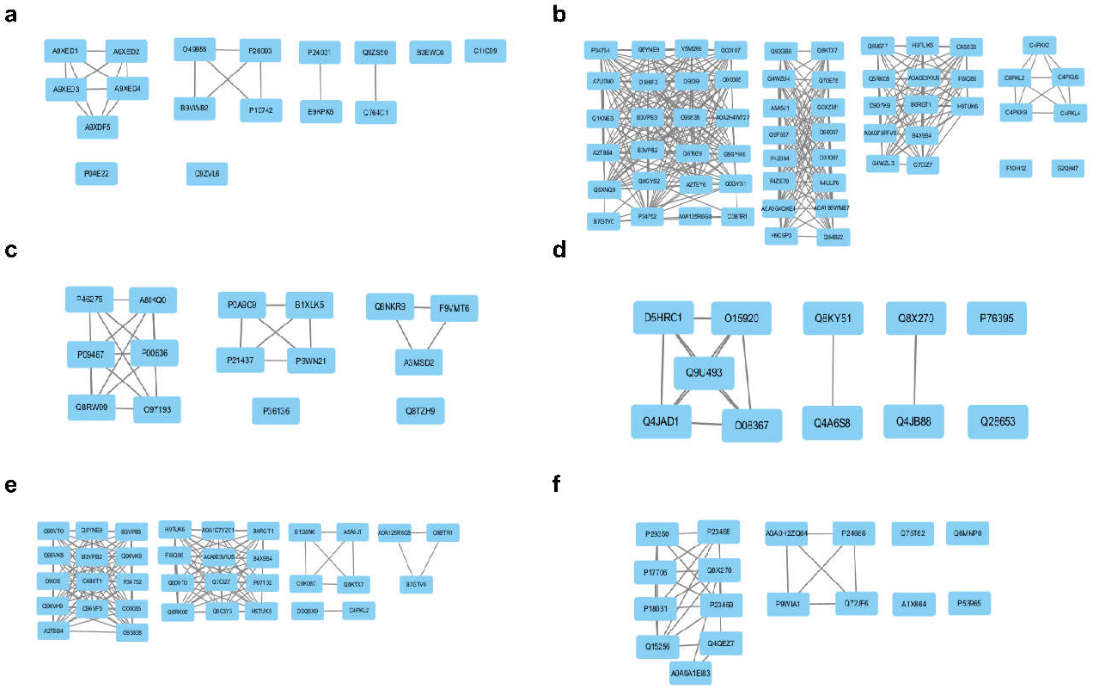

Figure S1. Sequence Similarity Network (SSN) for Different EC Number Groupings. Nodes represent phosphatase sequences. Edges represent similarity between two phosphatase sequences. **a.** 3.1.3.2. **b.** 3.1.3.8. **c.** 3.1.3.11. **d.** 3.1.3.16. **e.** 3.1.3.26. **f.** 3.1.3.48.

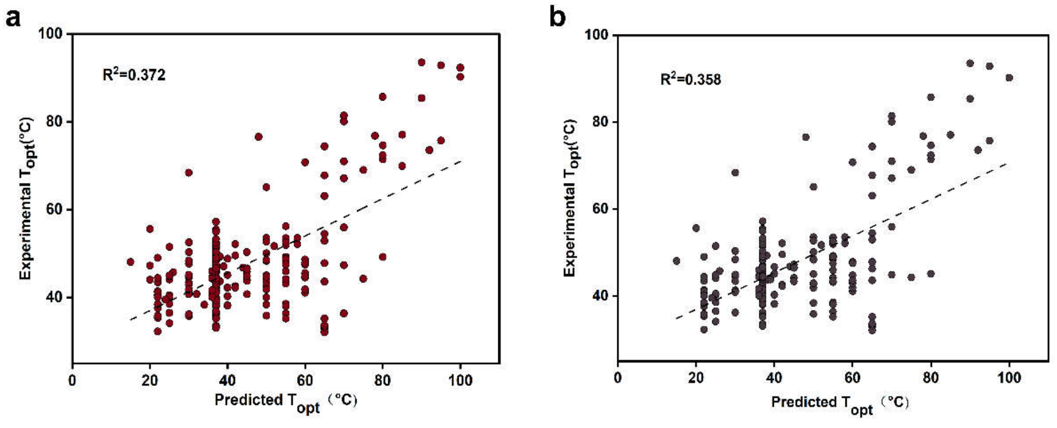

Figure S2. Prediction of T_{opt} values of phosphatases by DeepET. a. Comparison of predicted T_{opt} values versus experimental T_{opt} values on the T_{opt}249 phosphatase dataset. b. Comparison of predicted T_{opt} values versus experimental T_{opt} values on the T_{opt}218 phosphatase dataset.

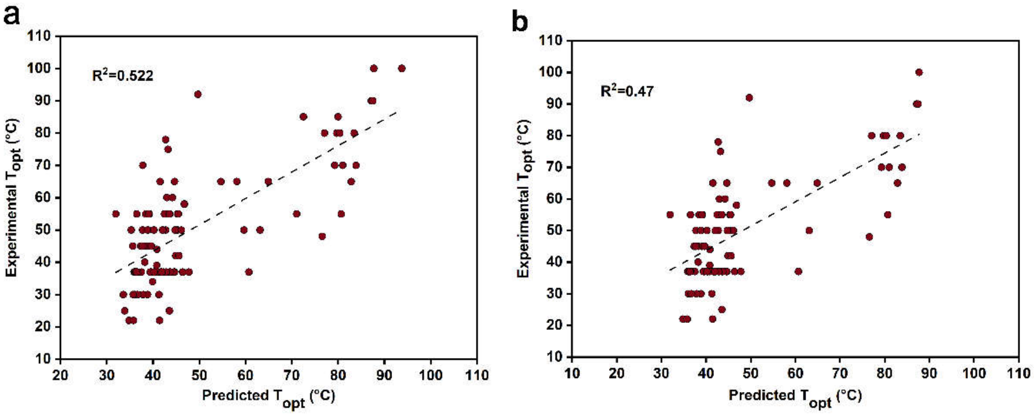

Figure S3. Prediction of T_{opt} values of phosphatases by TOMER. a. Comparison of predicted T_{opt} values versus experimental T_{opt} values on the T_{opt}116 phosphatase dataset. b. Comparison of predicted T_{opt} values versus experimental T_{opt} values on the T_{opt}100 phosphatase dataset.

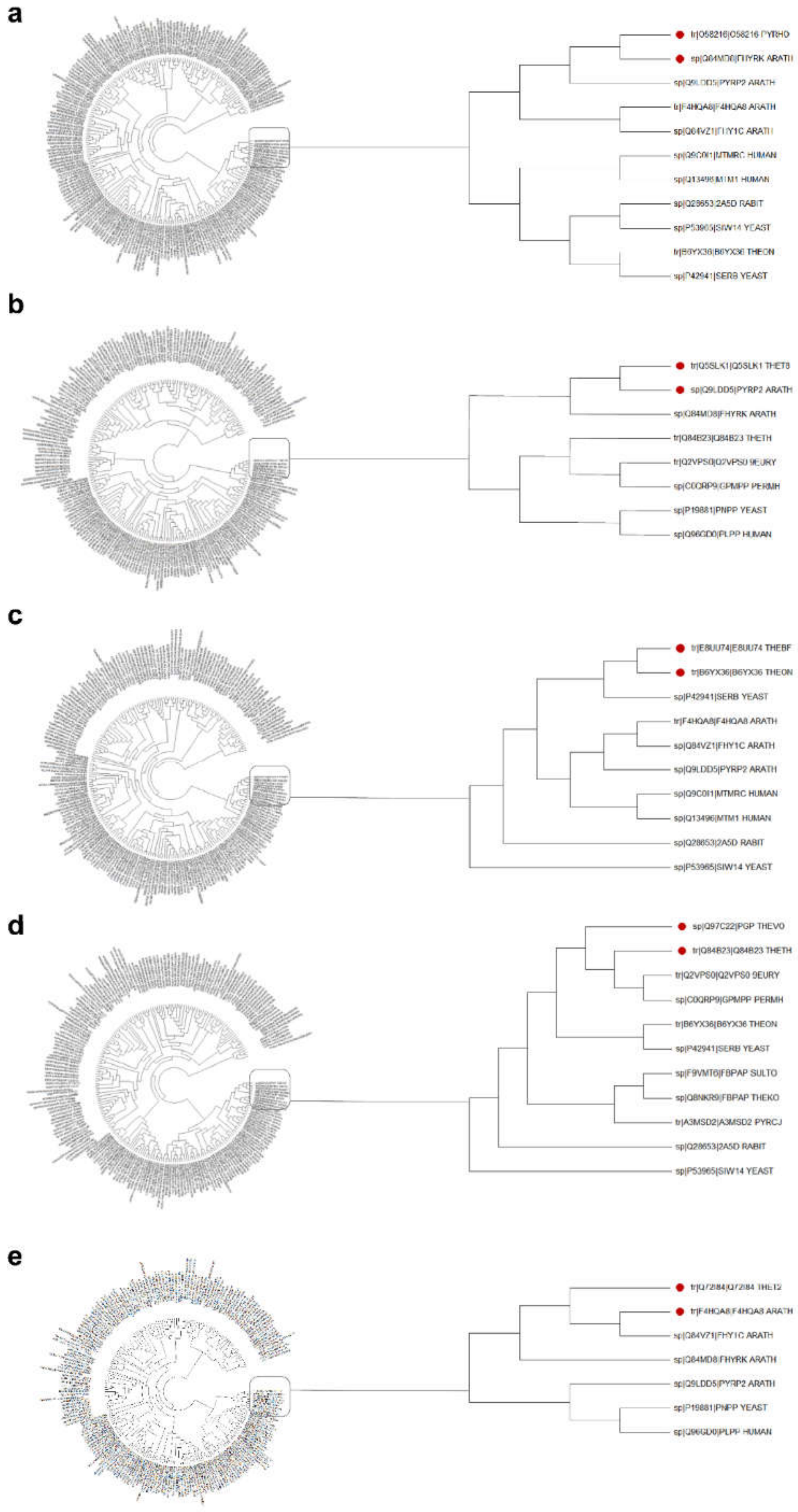
a
tr|O58216|O58216 PYRHO
sp|Q84MD8|FHYRK ARATH
sp|Q9LDD5|PYRP2 ARATH
tr|F4HQA8|F4HQA8 ARATH
sp|Q84VZ1|FHY1C ARATH
sp|Q9C0I1|MTMRC HUMAN
sp|Q13496|MTM1 HUMAN
sp|Q28653|2A5D RABIT
sp|P53965|SIW14 YEAST
tr|B6YX36|B6YX36 THEON
sp|P42941|SERB YEAST
b
tr|Q5SLK1|Q5SLK1 THET8
sp|Q9LDD5|PYRP2 ARATH
sp|Q84MD8|FHYRK ARATH
tr|Q84B23|Q84B23 THETH
tr|Q2VPS0|Q2VPS0 9EURY
sp|C0QRP9|GPMPP PERMH
sp|P19881|PNPP YEAST
sp|Q96GD0|PLPP HUMAN
c
tr|E8UU74|E8UU74 THEBF
tr|B6YX36|B6YX36 THEON
sp|P42941|SERB YEAST
tr|F4HQA8|F4HQA8 ARATH
sp|Q84VZ1|FHY1C ARATH
sp|Q9LDD5|PYRP2 ARATH
sp|Q9C0I1|MTMRC HUMAN
sp|Q13496|MTM1 HUMAN
sp|Q28653|2A5D RABIT
sp|P53965|SIW14 YEAST
d
sp|Q97C22|PGP THEVO
tr|Q84B23|Q84B23 THETH
tr|Q2VPS0|Q2VPS0 9EURY
sp|C0QRP9|GPMPP PERMH
tr|B6YX36|B6YX36 THEON
sp|P42941|SERB YEAST
sp|F9VMT6|FBPAP SULTO
sp|Q8NKR9|FBPAP THEKO
tr|A3MSD2|A3MSD2 PYRCJ
sp|Q28653|2A5D RABIT
sp|P53965|SIW14 YEAST
e
tr|Q72I84|Q72I84 THET2
tr|F4HQA8|F4HQA8 ARATH
sp|Q84VZ1|FHY1C ARATH
sp|Q84MD8|FHYRK ARATH
sp|Q9LDD5|PYRP2 ARATH
sp|P19881|PNPP YEAST
sp|Q96GD0|PLPP HUMAN

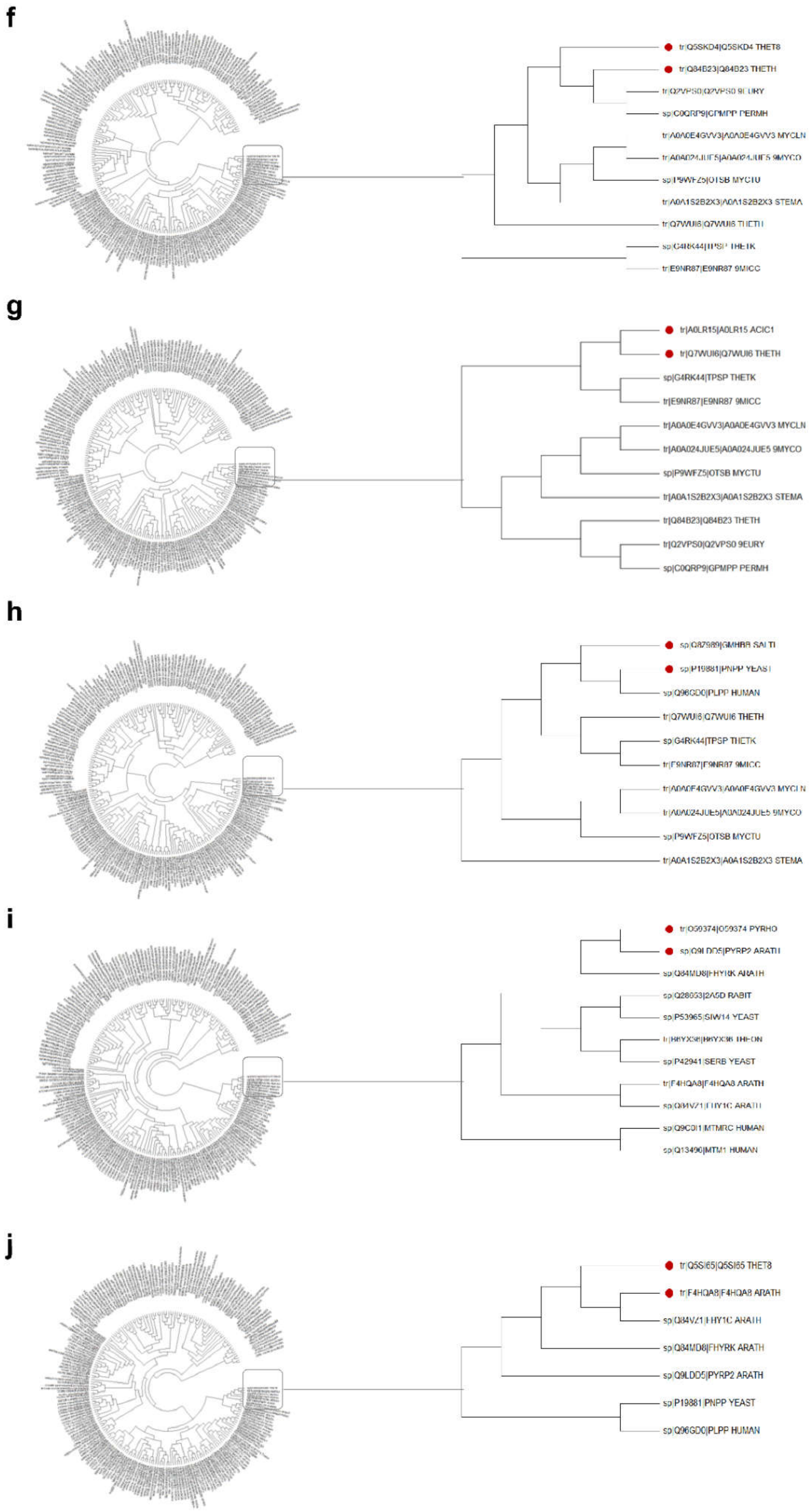

Figure S4. The evolutionary tree diagram of unkown phosphatase sequences. Highlighting the

target phosphatase and its closely related phosphatases. Nodes represent phosphatase sequences. Lines or arcs represent evolutionary relationships between sequences. **a.** O58216. **b.** Q5SLK1. **c.** E8UU74. **d.** Q97C22. **e.** Q72I84. **f.** Q5SKD4. **g.** A0LR15. **h.** Q8Z989. **i.** O59374. **j.** Q5SI65.

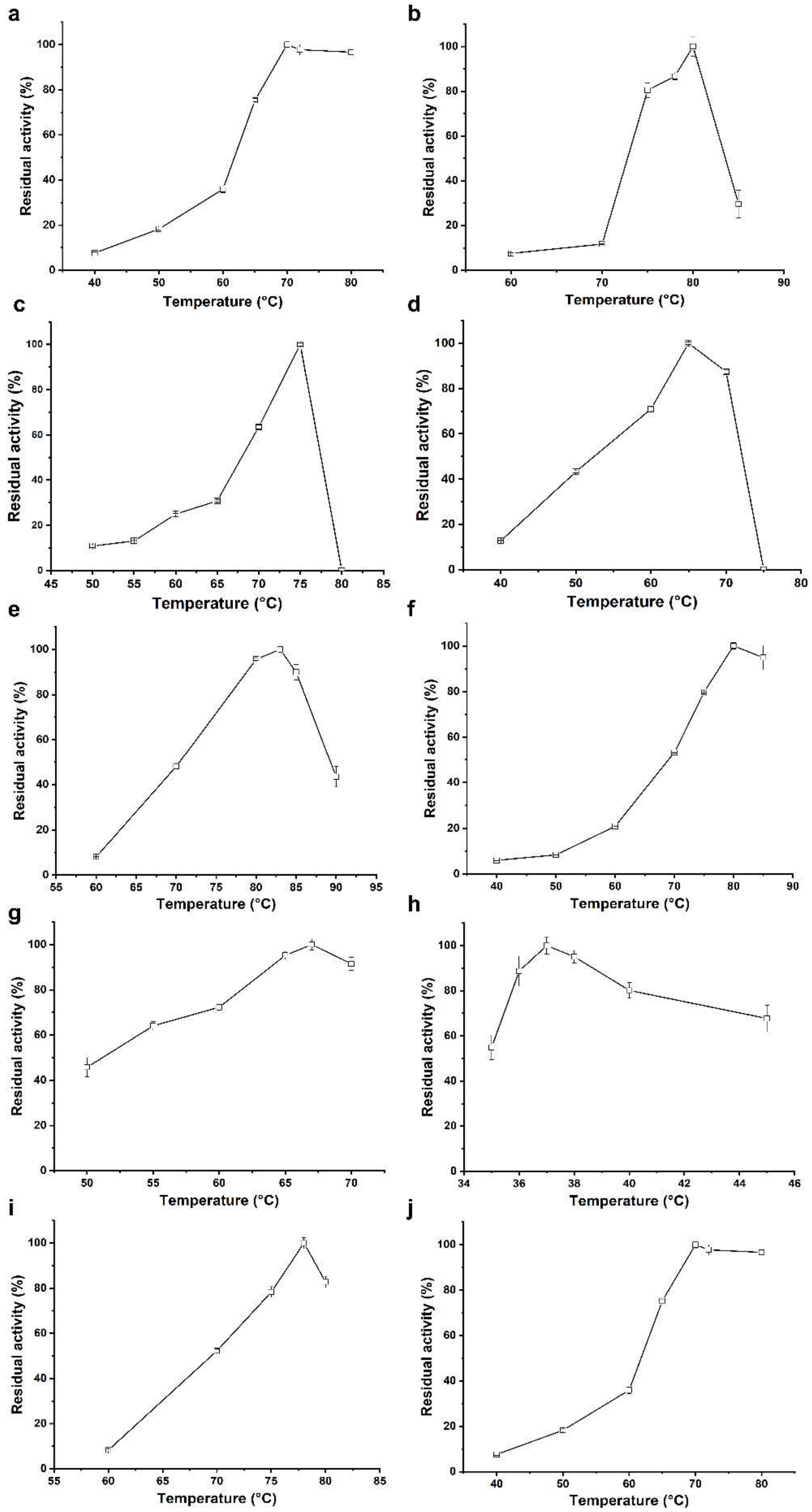

Figure S5. Experimental determination of T_{opt} values of phosphatases. **a.** Temperature profile of Phosphatase O58216. **b.** Temperature profile of Phosphatase Q5SLK1. **c.** Temperature profile of

Phosphatase E8UU74. **d.** Temperature profile of Phosphatase Q97C22. **e.** Temperature profile of Phosphatase Q72I84. **f.** Temperature profile of Phosphatase Q5SKD4. **g.** Temperature profile of Phosphatase A0LR15. **h.** Temperature profile of Phosphatase Q8Z989. **i.** Temperature profile of Phosphatase O59374. **j.** Temperature profile of Phosphatase O59374.

Table S1. Different EC numbers have optimal similarity thresholds.

EC number	Threshold
3.1.3.2	3
3.1.3.8	5
3.1.3.11	3
3.1.3.16	5
3.1.3.26	3
3.1.3.48	5
